# The Entropy Analysis Method for Assessing the Efficiency of Workload Distribution Among Medical Institution Personnel

**DOI:** 10.3390/e27050465

**Published:** 2025-04-25

**Authors:** Oksana Mulesa, Ivanna Dronyuk

**Affiliations:** 1 Department of Physics, Mathematics and Technologies, University of Presov, 080 01 Presov, Slovakia; oksana.mulesa@unipo.sk; 2 Department of Software System, Uzhhorod National University, 88000 Uzhhorod, Ukraine; 3 Department of Mathematics and Informatics, Jan Dlugosz University in Czestochowa, 42-217 Czestochowa, Poland

**Keywords:** entropy, data analysis, human resources, workload, quality of medical services, medical institution, healthcare system, labor optimization, efficiency assessment

## Abstract

The aim of this study is to develop a convenient and effective entropy analysis method for assessing the efficiency of workload distribution among medical institution personnel. This research is based on a model for evaluating employee workload in conditional time units—credits—taking into account time-and-motion studies and the volume of medical services provided or tasks performed over a given period. The model and method developed by the authors enable the consideration of potential losses of working time and coefficients that determine the percentage of effective working time. The method is based on calculating and analyzing the values of normative and actual workloads of employees. The study introduces such indicators as relative workload, workload distribution entropy, and the entropy of free and excessively worked time credits. During the experimental verification of the developed method for analyzing the activities of a dental clinic, it was demonstrated that the method is both convenient and effective for analyzing the performance of individual employees as well as groups of employees. The results of the method are presented in a convenient and intuitively understandable form. Therefore, this method can serve as an effective tool for identifying internal reserves within the institution and making managerial decisions regarding its further operation.

## 1. Introduction

The field of medical services is one of the priority areas of healthcare system activity. One of the goals of this activity is to provide high-quality, timely, and comprehensive medical services to all who need them [1]. The providers of medical services include both individual healthcare professionals and medical institutions as a whole. The processes involved in the operation of medical institutions are primarily related to the organization of medical service delivery and the execution of other tasks by the institution’s personnel. The efficiency of a medical institution’s activity can be assessed based on various indicators, ranging from the quantity and quality of medical services provided to the extent of coverage for all those in need [2,3].

The key figures in a medical institution are its personnel. The level of workload assigned to the staff directly affects the quality of services they can provide [4,5]. Therefore, one of the most interesting and important aspects of analyzing the efficiency of a medical institution’s personnel is the distribution of workload among its employees. The efficiency of workload distribution significantly influences many performance indicators of the institution, including the quantity and quality of services provided by employees, the level of population coverage, the allocation of available resources, compliance with labor regulations, and expected revenue, among others [6]. While analyzing workload distribution indicators in small medical institutions over a short period may be sufficiently handled by simple comparisons of numerical data, conducting a systematic analysis of workload distribution in institutions with a large number of employees of different specialties—who may provide various types of services and perform additional tasks—can be complicated by the large volume of data and its lack of systematization.

The aim of this study is to develop an entropy analysis method for assessing the efficiency of workload distribution among medical institution personnel, providing a clear and convenient tool for identifying productivity reserves, evaluating the uniformity of workload distribution, and more. Unlike traditional methods used to assess the workload of medical institution employees, our approach, which applies Shannon entropy and its modifications, enables the evaluation of the efficiency of information distribution regarding workload.

The main contribution of this paper can be summarized as follows:

- We performed an analysis and verbal-mathematical formulation of the problem of evaluating the efficiency indicators of a medical institution and its employees. The approach is based on the concept of time credit—a unit of time within which a particular service can be provided. Measuring services in standardized units allows for the comparison and consolidation of different services during analysis.
- We developed an entropy analysis method for assessing the efficiency of workload distribution among medical institution personnel, based on the concepts of normative and actual workloads of employees. Analyzing indicators derived from these values enables the identification of employee overload, reserves for workload redistribution, and other insights. The application of entropy in this method allows for the analysis of performance indicators across different personnel groups within the institution, making the method more user-friendly and informative.
- We conducted an experimental verification of the developed method using the case study of a dental clinic’s personnel activities.

The practical value of the developed tool lies in the standardization of approaches to analyzing the activities of employees across different categories involved in various tasks and service provisions. This approach can be applied in personnel activity analysis within medical institutions for internal audits and the development of managerial decisions regarding future institutional planning. This approach will improve human resource management in the healthcare sector and contribute to enhancing the quality of medical services by ensuring compliance with labor standards. An additional advantage of the method is that its results are presented in a convenient and intuitive form, and their interpretation does not require medical facility managers to have special mathematical skills.

The theoretical results of the study may serve as a foundation for solving optimization problems related to resource allocation in medical institutions and improving the efficiency of certain performance indicators.

The structure of the paper is as follows: The second section provides a review and critical analysis of scientific studies on the evaluation of medical institutions’ performance indicators, as well as the application of entropy in solving applied problems. The third section presents an analysis of the problem, a verbal-mathematical formulation of the workload assessment task for medical institution employees, the method developed by the authors, and the algorithm for entropy analysis to assess the efficiency of workload distribution among employees. The fourth section contains the experimental verification of the developed method using a case study of a dental clinic’s personnel activities. It demonstrates the application of the proposed method and examples of interpreting the results. The discussion and conclusions are presented in the final section.

## 2. Literature Review

The analysis of scientific sources related to productivity and efficiency assessment methods in medical institutions has shown that they can be divided into several main groups.

The first group consists of scientific sources that examine the analytical aspects of healthcare institution operations. In a previous study [7], an analysis was conducted on productivity assessment methods in the field of medical services. The authors reviewed various methods for evaluating qualified personnel based on their usefulness in the workplace. The study revealed that none of these methods have a unified methodology and are highly influenced by subjective factors. Only the combination of different methods can provide a more objective result. Thus, the study highlights the necessity of using multifactor approaches to assessing productivity in the healthcare sector. For example, in a previous study [8], an analysis was conducted on the impact of hospital structure and organizational restructuring on nursing personnel. Based on a literature review, the authors substantiated that the positions nurses hold within a medical institution influence their ability to provide proper and safe care. Studies [9,10,11] examined the application of artificial intelligence in healthcare management. The authors demonstrated that modern developments in mathematics and intelligent analysis enhance the effectiveness of managerial decisions in healthcare organizations.

A separate group consists of studies dedicated to assessing the efficiency of institutions with different specializations and their personnel. For example, in previous studies [12,13], models for measuring key performance indicators in higher-education institutions were developed. The proposed models facilitate the measurement and explanation of an institution’s success through multiple dimensions. In other studies [14,15], the experience of non-profit organizations in evaluating their efficiency was examined. However, these approaches are primarily focused on final performance outcomes rather than on balancing the workload among employees. They do not take into account the uniformity of workload distribution or potential time reserves, which are critical for the healthcare sector.

Furthermore, in healthcare, analytical methods are used to assess resource allocation. The most commonly applied methods include Data Envelopment Analysis (DEA), stochastic modeling, optimization approaches, and artificial intelligence methods. These methods are widely used for both resource allocation optimization and efficiency assessments of medical institutions and individual employees. The most common method for assessing the technical and allocative efficiency of medical institutions and their departments is DEA [16,17]. This method is effective for comparing the efficiency of different Decision-Making Units (DMUs) (structural units or individual employees) by analyzing their productivity relative to the best-performing counterparts. It is based on the principle that an organization is considered efficient if it can achieve the maximum output (e.g., the number of patients treated) while using a given combination of input resources. Alternatively, an organization is efficient if it produces a certain level of output while using the minimum possible input resources. This approach is effective for resource allocation optimization and identifying underperforming departments. However, DEA has certain limitations in balancing workloads among personnel. For example, if an additional parameter is included in the model to represent labor standards, it would allow for assessing whether an employee’s workload complies with established norms. However, the maximum workload threshold and the method for its calculation remain open questions, requiring further modeling. Additionally, DEA does not provide information in time units, which complicates its interpretation and application for real-time management.

Artificial intelligence methods, particularly reinforcement learning-based deep learning, are also used to automate resource management in healthcare. For example, in a previous study [18], the authors investigated the problem of distributing limited healthcare resources. They demonstrated that their developed method could improve both efficiency and fairness in healthcare resource allocation. Stochastic modeling is also used to forecast the workload of medical personnel [19]. This study proposed an integrated approach that combines deep learning and stochastic optimization for resource management in team-oriented medical systems. However, these models also do not provide precise quantitative estimates in time units, making them challenging to apply for real-time workforce management. Additionally, despite the effectiveness of AI-based methods, a significant limitation is their complexity and the requirement for large datasets. In a previous study [20], the authors developed a healthcare resource allocation rule that respects priorities, meets acceptability requirements, distributes the maximum possible number of units, and does not incentivize agents to conceal their qualification category. The rule is based on set theory and binary relations, ensuring that resources are allocated as efficiently as possible, with no unused resources left. This solution enables efficiency, fairness, and optimal resource utilization simultaneously. However, despite its high level of formal optimization, this method does not address workload balancing among medical personnel.

Additionally, to analyze the performance indicators of medical institutions, methods such as data analysis, business logistics, and queueing theory are commonly used [21,22,23]. However, the approaches described above do not fully address the issue of service quality. Maximizing the efficiency of human resource utilization may lead to violations of labor standards and burnout among medical personnel.

Thus, the development of approaches that consider not only resources but also the uniformity of workload distribution within medical institutions, do not require large datasets, and allow for identifying internal reserves of medical institutions to improve their efficiency is highly relevant. Additionally, an important advantage of such approaches should be the presentation of results in a convenient and intuitively understandable form, ensuring effective real-time management. From this perspective, methods based on the concept of entropy analysis are of particular interest.

Shannon entropy has been successfully used for analyzing the uniformity of resource distribution in various systems.

For example, in a previous study [24], an approach was developed for cyberattack protection by identifying potential attack vectors based on entropy analysis of cybersecurity events. The study results confirmed that such entropy-based analysis detects security threshold violations and anomalies in operating systems and applications, thereby revealing potential attack vectors. In his research [25], Bernard Twaróg applied Shannon entropy to analyze long-term climate variability, focusing particularly on precipitation and temperature. The study confirmed the usefulness of Shannon entropy in climatological research and emphasized its potential for improving our understanding of complex and chaotic climate systems. Studies [26,27] have also demonstrated successful cases of applying entropy-based methods in medical diagnostics of specific diseases, proving their effectiveness in clinical medicine. The authors of other studies [28,29] applied information theory with entropy as a central concept for assessing the efficiency and costs of organizational structures and coordination processes in medical institutions. The main conclusion drawn was that combining correlation perspectives with entropy theory provides a quantitative assessment of how medical institution operations can be structured.

Thus, it can be concluded that the application of entropy analysis is an effective tool for evaluating the uniformity of parameter distribution in complex systems. This confirms its potential in healthcare, particularly for analyzing workload balancing among medical personnel.

This study presents a new entropy analysis method for assessing the efficiency of workload distribution among medical institution personnel, based on the concept of time credits. This approach allows for:

- Standardizing medical services and tasks performed within a medical institution and comparing the activities of different employee categories within a unified workload assessment system.
- Comparing the performance of various categories of medical personnel based on objective indicators of workload distribution uniformity.
- Identifying workload imbalances and assessing overloaded or underutilized personnel in time units.

The method is based on the concept of ensuring and maintaining the quality of medical services, which can be achieved by comparing the volume of time credits required for the high-quality provision of medical services with the actual time credits available to a medical worker within their working hours. This distinguishes the proposed method from those analyzed above. The application of the developed method provides a simple and convenient tool for the real-time analysis of medical institution employees’ performance, as well as making managerial decisions regarding the current organization of the institution’s operations and its future development planning.

## 3. Materials and Methods

### 3.1. Problem Analysis and Verbal-Mathematical Formulation of the Task

This study examines the problem of analyzing the performance efficiency indicators of medical institution personnel. The efficiency of medical institutions can be assessed through the analysis of various indicators, including the quality of medical services, staff competence, and the number of service refusals, among others.

The focus of this research is on the relationship between the actual work performed, services provided, and the normative workload of employees. The study is based on the assumption that all medical institution employees are qualified and provide only those services that correspond to their specialty, qualification, and position. Clearly, assessing the efficiency of working time utilization cannot be limited to the volume of services provided or tasks completed, as different services require varying amounts of time for their delivery. Following the approach in previous studies [30,31], which proposed models for evaluating the potential of medical institutions based on time credits—the smallest conditional time units required to provide a medical service—each medical service will be characterized by the number of time credits needed for its provision. To enable the analysis of the relationship between normative and actual workload, each employee will be assigned a corresponding number of time credits available during the analyzed period.

At first glance, it may seem that improving the efficiency of a medical institution simply requires increasing the number of services provided by its employees. However, given the specifics of the medical field, it can be stated that for each employee, there exists a threshold beyond which increasing the number of services provided within a given period may lead to a decline in their quality. This can be attributed to the physical and emotional exhaustion of employees, as well as having insufficient time to perform all necessary procedures properly [6,32,33]. Therefore, we will assume that a service provided by medical institution employees is considered high-quality if it is delivered by a qualified specialist or a team of specialists within an adequate time frame.

Thus, we will further consider the problem of analyzing medical personnel efficiency indicators as the problem of analyzing the relationship between the normative and actual workloads of employees over a given period. In institutions with a large number of employees of varying qualifications and specialties, conducting a continuous detailed analysis of each individual employee’s activity is complex and resource-intensive. Therefore, the authors propose an approach in which an initial analysis of the institution as a whole is advisable for identifying workload imbalances or internal reserves. If discrepancies between normative and actual workloads are detected, the next step would be to analyze the specific performance indicators of individual employees.

According to the general rule, the analysis of a medical institution’s performance is based on the relationship between its potential and the actual medical services provided. However, this approach does not allow for tracking individual employee overloads or identifying staffing reserves within the institution. To enable the analysis of individual employees’ performance and facilitate effective managerial decision-making based on this analysis, the authors propose the following verbal-mathematical formulation of the problem.

Let us consider a hypothetical medical institution. To construct the verbal-mathematical formulation of the efficiency assessment problem, we introduce the following notations:

N—the number of employees in the institution who provide services and perform tasks.

M—the number of different types of services and tasks that can be performed by the institution’s employees.

T—the period under study (in working days).

$S=(s_{1},s_{2},\ldots ,s_{M})$—a vector characterizing the volume of time credits required for service provision, where $s_{j}$ is the number of time credits per employee necessary for providing a service with index j, $j=\bar{1,M}$.

$ER=(er_{1},er_{2},\ldots ,er_{N})$—a vector characterizing the workload shares of employees, where $er_{i}$ is the workload share in the institution for the employee with index i, $i=\bar{1,N}$.

$WQ=(wq_{1},wq_{2},\ldots ,wq_{N})$—a vector whose components characterize the qualification of employees.

$X_{T}={\left\{{\bar{x}}_{i}=(x_{i1},x_{i2},\ldots ,x_{iM})\right\}}_{i=\bar{1,N}}$—a set of vectors whose components contain information about the actual work performed and services provided by employees of the medical institution, where $x_{ij}$ represents the number of services with index j actually provided by employee i over the period T.

It is necessary, based on the described input data, to determine the key indicators of employee performance efficiency in the medical institution. These indicators include the following:

- Normative workload of an employee.
- Actual workload of an employee.
- Relative workload of an employee.
- Entropy of workload distribution among employees.
- Difference between actual and normative workloads of employees.

Analyzing these indicators will provide insights into how effectively medical institution employees are engaged in performing their tasks.

Next, the authors propose a developed method that enables the assessment of efficiency at both the individual employee level and the institution as a whole.

### 3.2. Entropy Analysis Method for Assessing the Efficiency of Workload Distribution Among Medical Institution Personnel

The entropy analysis method developed by the authors consists of the sequential implementation of the following three stages:

Stage 1. Assessment of the normative workload of medical institution employees.

Stage 2. Assessment of the actual workload of medical institution employees.

Stage 3. Analysis of the relationship between normative and actual workloads of medical institution employees.

In developing this method, the authors utilized the concept of entropy, which allows not only for the examination of absolute and relative values but also for assessing their uniformity. This approach, in the authors’ view, provides a more effective tool for analyzing the performance of a medical institution.

We will now specify each of the outlined stages.

#### 3.2.1. Assessment of the Normative Workload of Medical Institution Employees

To assess the normative workload of medical institution employees ($NB_{i}$), we introduce the function of the following form (1):

(1) $$NB=\left(\phi (wq,er)-\chi (wq,er)\right)\cdot T,$$

where ϕ(wq,er) is a function for calculating the maximum number of credits an employee with qualification wq and workload share er possesses per working day.

χ(wq,er) is a function for calculating the average losses of working time associated with possible business trips, temporary incapacity for work, qualification improvement, etc.

The function ϕ(wq,er) is usually defined analytically, depending on the specifics of the medical institution’s operation and national legislation. An example of such a function is given by equation:

(2) ϕ(wq,er)=B(wq)⋅er⋅η(wq,er),

where B(wq) is a coefficient representing the total number of credits (e.g., minutes of working time) that an employee with qualification wq, working under full-time employment, possesses in the institution. This coefficient is determined by the employment contract or the medical institution’s staffing schedule.

η(wq,er) is a function that defines the share of effective working time for an employee with qualification wq and workload share er, taking values in the interval (0;1). It is important to note that effective working time refers to the time that an employee spends directly on providing services. This function allows for the exclusion of time spent on documentation, work-related discussions with colleagues, and similar activities. The structural and parametric identification of the function η(wq,er) can be performed based on expert surveys or time-and-motion studies when observing employee working hours at the institution. Depending on the healthcare system, this process may be based on regulatory acts or methodological guidelines [34,35,36]. The value of this function will depend on the specifics of the medical institution and the healthcare system in which it operates. For instance, according to a study conducted in Australia in 2007, the effective working time for nurses accounted for approximately 47% of their total working time [37]. In contrast, in Belgium, a study conducted in 2015–2016 found that the share of effective working time for nurses was 30.78% [38].

In turn, the function χ(wq) is typically identified considering statistical data on working time losses and the results of surveys of employees with the corresponding qualifications, regarding expected business trips, qualification improvements, etc., for future periods. In a previous study [39], a two-stage identification method was presented based on survey results from a target group and expert evaluations. According to the proposed methodology, the first stage involved surveying representatives of the target group, who, based on their experience, indicated the average losses of working time. The responses obtained formed the basis for the expert questionnaire in the second stage. In the second stage, qualified experts—healthcare administrators—provided their assessments and conclusions.

Thus, the algorithm for the method of assessing the normative workload of institution employees involves identifying the components that form the basis of function (1).

#### 3.2.2. Assessment of the Actual Workload of Medical Institution Employees

To assess the actual workload of medical institution employees ($RB_{i}$), it is necessary to correlate the volumes of completed tasks and medical services provided with the time credits allocated for such services. Thus, the actual workload of an employee can be calculated using the following Formula (3):

(3) $$RB_{i}=\sum_{j=1}^{M}s_{j}x_{ij}$$

It should be noted that in real conditions of a medical facility, depending on the complexity of the clinical case or, for example, the occurrence of emergency situations, some services may require more time than is planned in standard time measurements. In such cases, the medical worker who provided the service is recommended to record it in the corresponding component of the vector ${\bar{x}}_{i}$ with a duration coefficient, which, when multiplied by the standard time assigned to the service, shows its actual duration. This approach ensures the flexibility of the model in cases of unusual or complicated situations during the provision of medical services.

#### 3.2.3. Quantitative Analysis of the Relationship Between Normative and Actual Workloads of Medical Institution Employees

The assessment of the efficiency of medical institution employees is proposed to be conducted through the analysis of a set of indicators calculated based on the normative and actual workload values determined in the previous stages. Let us consider some of these indicators and the conclusions that can be drawn from their values.

An important indicator of the efficiency of an employee’s working time utilization is the ratio of actual workload to normative workload. We introduce the concept of the relative workload of an employee ($p_{i}$), which is calculated using Formula (4):

(4) $$p_{i}=\frac{RB_{i}}{NB_{i}},\;\;i=\bar{1,N}.$$

From the previous discussion, it follows that $p_{i}$ is a non-negative real number. Let us consider possible cases and conclusions that can be drawn from them:

1. If $p_{i}=1$, this means that employee i is utilizing their working time efficiently.
2. If $p_{i}>1$, then the employee performs more tasks and provides more services than required to ensure their proper quality. This means that the employee was overloaded during period T.
3. If $p_{i}<1$, then employee i was underloaded during period T.

Next, based on the relative workload of employees, it is possible to analyze the overall performance of the medical institution using the entropy of workload distribution, which is calculated using Formula (5):

(5) $$H=-\sum_{i=1}^{N}{\hat{p}}_{i}\log_{2}({\hat{p}}_{i}),$$

where ${\hat{p}}_{i}$ is the normalized value of the relative workload of employee i, calculated using Formula (6):

(6) $${\hat{p}}_{i}=\frac{p_{i}}{\sum_{l=1}^{N}p_{l}},\;\;i=\bar{1,N}$$

It is understood that $H\in \left[0;\log_{2}(N)\right]$. The closer the value of H is to the maximum ($H_{\max}=\log_{2}(N)$, the more evenly distributed the workload among employees of the institution.

The entropy indicator of workload distribution among employees, unlike the commonly used indicator that analyzes the ratio of total actual workload to total normative workload ($\sum_{i=1}^{N}RB_{i}/\sum_{i=1}^{N}NB_{i}$), allows for the identification of workload distribution imbalances among employees.

Similarly, analyzing the performance of employees within a specific category enables the identification of workload redistribution reserves when needed. In this case, for employees with qualification $wq^{*}$, the following calculations should be performed:

(7) $${\hat{p}}_{i}=\frac{p_{i}}{\sum_{l=\bar{1,N}:\;wq_{l}=wq^{*}}p_{l}},\;\;i\in \{1,2,\ldots ,N\}:\;wq_{i}=wq^{*}$$

(8) $$H_{wq^{*}}=-\sum_{i=\bar{1,N}:\;wq_{i}=wq^{*}}{\hat{p}}_{i}\log_{2}({\hat{p}}_{i})$$

The next indicator of medical institution employee performance efficiency is the difference between actual and normative workloads, which is calculated using Formula (9):

(9) $$R_{i}=\left\{\begin{array}{l} NB_{i}-RB_{i},\;\;\;if\;\;NB_{i}>RB_{i},\\ 0,\;\;otherwise, \end{array}\right.\;\;\;i=\bar{1,N}.$$

As seen in rule (9), the indicator $R_{i}$ allows for identifying time credit reserves only among underloaded employees.

The indicator $R_{Total}$, calculated using Equation (10), makes it possible to determine the institution’s overall potential available for redistributing services as well as for introducing new tasks or services:

(10) $$R_{Total}=\sum_{i=1}^{N}R_{i}$$

To analyze the uniformity of time reserves among employees, similar to (5) and (8), entropy is proposed to be applied as follows:

(11) $$H_{R}=-\sum_{i=\bar{1,N}:\;R_{i}\neq 0}{\hat{R}}_{i}\log_{2}({\hat{R}}_{i})$$

where ${\hat{R}}_{i}$ is calculated using (12):

(12) $${\hat{R}}_{i}=\frac{R_{i}}{\sum_{l=\bar{1,N}:\;R_{l}\neq 0}R_{l}}$$

A maximum value of $H_{R}$ indicates an even distribution of time reserves. If $H_{R}$ is close to 0, it suggests that some employees in the institution have significantly larger free time reserves compared to others.

Similarly to (7) and (8), it is also possible to analyze time reserves for specific categories of employees and examine the volumes of excessively worked time credits, which we will denote as $H_{O}$.

#### 3.2.4. Illustration of Possible Scenarios for Analyzing Human Resources in a Medical Institution

Let us present several scenarios for analyzing the developed indicators and the conclusions that can be drawn from them. These scenarios can be applied both to the institution as a whole and to specific employee groups.

Case 1. If $H∼H_{\max}$ and $\sum NB_{i}\ll \sum RB_{i}$, this potentially indicates a significant overload of most employees. One way to address this issue is to hire new staff.

Case 2. If $H∼H_{\max}$ and $\sum NB_{i}\gg \sum RB_{i}$, this indicates a systematic underload of the institution’s employees. In this case, the institution has significant human resource potential to provide new services or attract more clients.

Case 3. If $H_{R}∼0$ and $R_{Total}\geq \frac{1}{N}\sum NB_{i}$, this indicates that at least one employee is significantly underloaded. If necessary, this employee can be fully relieved from duties, and their workload can be redistributed to reduce the burden on other employees.

Case 4. If $H_{R}∼0$ and $H_{O}∼0$, significant redistribution of responsibilities within the studied employee group may be carried out to enhance workload balance and reduce overload among individual employees.

The described scenarios for human resource analysis are not exhaustive and may be modified.

### 3.3. Algorithm for Entropy Analysis of the Efficiency of Workload Distribution Among Medical Institution Personnel

Based on the formulated verbal-mathematical problem statement and the method developed by the authors, the following algorithm was designed for a specific medical institution:

Step 1. Calculation of normative workloads of employees.

Step 2. Calculation of actual workloads of employees.

Step 3. Comparison of the total actual and normative workloads of employees to identify overall reserves within the medical institution or determine the need to increase staff numbers.

Step 4. Calculation of workload distribution entropy among employees.

Step 5. If the entropy value significantly deviates from the maximum, calculate workload distribution entropy for specific employee categories to identify those with uneven workload distribution.

Step 6. Analysis of employees whose actual workload exceeds the normative workload.

Step 7. To identify time credit reserves, calculate the entropy of the distribution of time credit reserves and excess credits both for the institution as a whole and for specific employee categories.

Step 8. Apply scenarios for developing managerial decisions.

The step-by-step application of this algorithm enables medical institutions to conduct an effective ongoing analysis of both individual employees and the institution as a whole.

## 4. Results

### 4.1. Collection of Data

For the experimental verification of the developed method and demonstration of its effectiveness, we selected a private dental clinic located in Uzhhorod, Ukraine. The clinic employs 25 staff members:

- 2 orthodontists.
- 2 surgeons.
- 4 therapists.
- 2 hygienists.
- 10 nurses.
- 2 administrators.
- 3 sanitation workers.

The clinic provides 24 different medical services. For each service, time-and-motion studies were conducted to determine the number of credits (minutes) required for its high-quality execution. If multiple employees were involved in providing a service, separate time credits were assigned for each category of staff. The data on services and time credits are presented in Table A1.

In the next stage, expert surveys and statistical data analysis of the clinic’s operations were used to identify the parameters of the functions ϕ(wq,er) and χ(wq,er). The summarized results are presented in Table 1.

### 4.2. Verification of the Developed Method

To simplify the verification process and its detailing in this study, the analysis will be performed only for medical services and medical staff. The verification process follows the steps of the algorithm described earlier. The base period selected was one workweek, which, under full-time employment conditions, consists of 40 working hours.

Based on the identified parameters of the functions ϕ(wq,er) and χ(wq,er), and considering the employment share, the normative workload was calculated for each medical employee using Formulas (1) and (2). Adhering to this workload ensures high-quality medical services and helps prevent professional burnout and other negative consequences of employee overload.

To calculate the normative workload of employees, statistics on the services provided by staff over the analyzed workweek were used. The actual workload was calculated using Formula (3).

**Figure 1:**
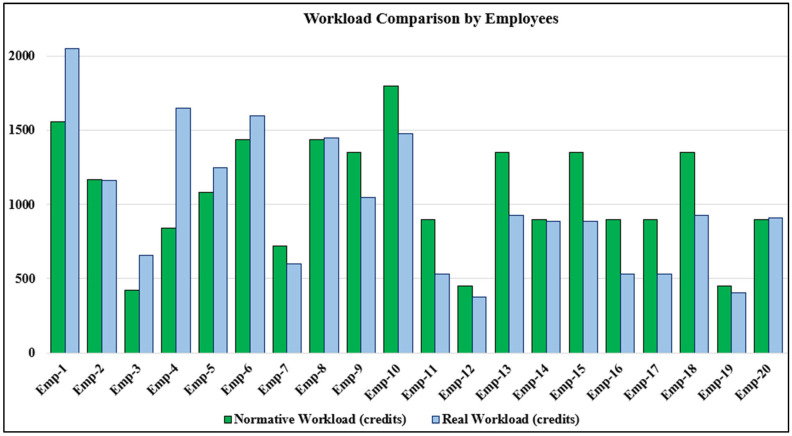
Comparison of normative and real workload (credits) across employees.

The normative and actual workloads of each medical employee are presented in Table 2 and Figure 1.

**Figure 2:**
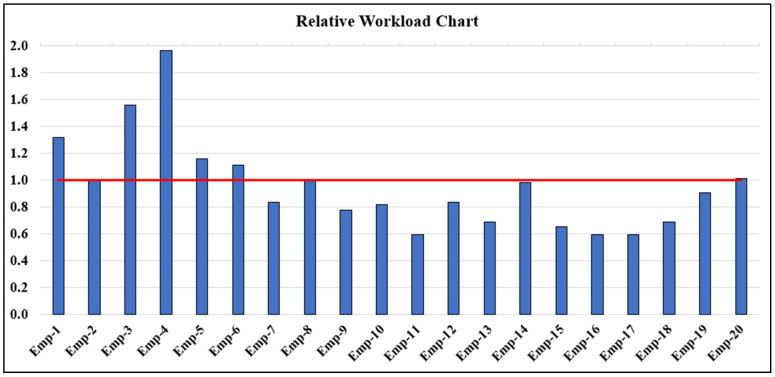
Relative workload distribution of employees with a benchmark level.

At the stage of comparing actual and normative workloads, the relative workloads of employees were calculated. The relative workload diagram of medical employees is presented in Figure 2, where the red line represents the reference norm, indicating the full actual workload for an employee.

To understand the structure of free time credits, we analyze their entropy both for all medical staff in the institution and for specific employee categories. The calculation results are presented in Table 3.

In the next stage, we analyze the structure and distribution of excessively worked time credits. The calculation results are presented in Table 4.

For convenience, Table 3 and Table 4 include percentage ratios of entropy values to their maximum possible values (fifth column in each table).

## 5. Discussion

In the experimental part of the study, we demonstrated the application of the developed model and method using the example of analyzing the work of medical personnel in a dental clinic. For the defined list of services, time tracking was conducted (Table A1), and calculations were performed based on the completed tasks. Thus, the diagrams in Figure 1 and Figure 2 clearly visualize the relationship between actual and normative workloads for all employees. Based on these, in cases where there is a small number of employees, useful information can be obtained regarding the overload or underload of specific workers (for example, Emp-4 performs an actual workload nearly twice as high as the normative workload, while Emp-2, Emp-14, and Emp-20 perform within their expected workload range).

Based on the results presented in Figure 2, conclusions can be drawn regarding the underutilization or overloading of certain employees. As seen in Table 2, the clinic’s normative capacity is 21,270 credits, while the actual workload completed was 19,852 credits. Since some employees have an actual workload exceeding their normative workload, it can be concluded that the medical staff of the clinic have more than 1418 free time credits.

Regarding group-level analysis, for example, from the data in Table 3, we can conclude that among the two underloaded doctors, one has more free time than the other, while in the group of hygienists, both are evenly underloaded (relative entropy 39%).

From Table 4, we see that surgeons experience the highest level of overwork, with an uneven distribution of excess workload (76.9% relative entropy), which may indicate significant overload for one of them.

Table 3 also shows that the medical staff collectively have 3301 free time credits. The largest number of unused credits belongs to nurses (2546 credits), with a relative entropy of 88.6%, indicating a highly uneven distribution of free credits. Meanwhile, the hygienists have 625 free credits with an almost uniform distribution (relative entropy of approximately 99.9%).

From Table 4, we see that, in total, the clinic’s employees have worked 1883 excess credits, with a moderately uniform distribution (74.5%). The smallest excess workload belongs to one nurse (8 excess credits). Surgeons have worked 1045 excess credits, with overload distribution between the two employees being moderately uneven (76.9%).

Further analysis of the obtained results allows us not only to determine the level of overload or underload of individual employees but also to make effective managerial decisions regarding the organization of work and the optimization of human resource distribution in a medical institution. The proposed method enables the assessment of workload distribution efficiency among the institution’s personnel, as well as its detailed evaluation.

An important advantage of this method is its ease of interpretation. Unlike other efficiency assessment methods that operate with relative coefficients or complex optimization models, our method presents the results in a clear and convenient format—in time credits (hours or minutes). This approach will allow medical institution managers to have continuous access to understandable information about the current state of workload distribution among employees and, based on this, to make managerial decisions regarding, for example, redistributing workload, hiring additional staff, retraining existing employees, or expanding the range of services provided by the institution’s employees.

It should also be emphasized that the use of the method we developed does not require healthcare managers to have special knowledge of optimization or entropy theory. The indicators, which are calculated in time credits, are intuitively understandable. The entropy values, in turn, serve as indicators of how evenly the workload is distributed. That is, the closer the value is to the maximum possible, the more balanced the distribution is. All of this makes the method accessible and useful for medical facility managers and allows them to make informed management decisions in a convenient format.

The entropy analysis method complements existing econometric approaches, such as DEA and stochastic modeling, by adding a tool for assessing workload distribution uniformity and identifying time-related needs or reserves. The proposed approach enhances the capabilities of these methods by providing healthcare management with an additional measure of workload efficiency—work–time balance.

Using an entropy-based uniformity measure allows us not only to detect overloaded or underutilized employees but also to identify workload imbalances within specific groups. Analyzing these indicators will help uncover internal workforce reserves within the institution.

Directly based on the results obtained using the method developed in this study, the following managerial decisions can be made:

- Redistribution of tasks among employees to prevent overload.
- Retraining employees (where applicable) in case of imbalances between different specializations.
- Expanding the range of services provided by the institution’s employees if internal time reserves are available.
- Hiring new employees in case of systematic overload of existing staff, etc.

The results of this study align with modern trends in evaluating the efficiency of resource utilization, including human resources, in medical service delivery. As demonstrated in previous studies [6,32,33], eliminating burnout caused by excessive workload will increase the resilience of medical institutions and the healthcare system as a whole. The method we have developed can serve as a convenient, employee-oriented tool for preventing a decline in medical service quality that may arise due to violations of labor standards.

The proposed method is scalable and can be applied to analyze the operations of medical institutions of any size. Additionally, the method can be used not only for evaluating the performance of individual doctors but also for analyzing specializations or medical services, which can serve as an effective tool for planning operations and workforce policies in healthcare institutions.

Thus, it can be concluded that tools focusing not only on overall productivity indicators but also on workload distribution uniformity are crucial for evaluating the efficiency of medical personnel and institutions as a whole. These tools can be a key factor in ensuring a sustainable healthcare management system that guarantees both operational efficiency and a comfortable work pace for personnel.

## 6. Conclusions

The research results indicate the importance and relevance of the developed method for assessing the efficiency of workload uniformity among medical institution employees. Based on the results obtained during its application, it is possible to identify workload distribution imbalances among medical institution employees and detect internal reserves for workload redistribution or the introduction of new services. Additionally, the method allows for identifying cases of significant overload among individual employees. The method is convenient to use as it provides results in a form that is easy to analyze and interpret.

The experimental verification conducted using a dental clinic as an example confirmed that the method effectively analyzes both individual employees and their groups. The results have been shown to serve as a basis for determining staffing needs, analyzing resource allocation efficiency, and developing optimal work organization models. A particularly important aspect is the ability to apply the method not only at the level of individual employees but also for specializations or medical services, providing additional grounds for managerial decision-making in the healthcare sector.

The main limitation of this method is the need to determine time tracking (chronometry) for individual services. This process can be quite labor-intensive and must be implemented during the setup phase for a specific type of medical institution. However, under certain conditions, to simplify this process, the time tracking of individual medical services can be determined through expert surveys or based on an analysis of available statistical data.

The uniqueness of the method lies in its focus on optimizing and establishing a uniform workload distribution among medical personnel while ensuring the quality of services provided and preventing physical overload of employees. This is implemented in contrast to other methods, which are primarily focused on productivity and efficiency from the patient’s perspective. The proposed method complements existing resource efficiency assessment methods and allows for more flexible management of human resources in medical institutions. The method is intuitively understandable and does not require users to have special mathematical training. All of this makes it an effective tool for making well-grounded management decisions in the field of healthcare.

Further research may be aimed at analyzing the dynamics of changes in the workload of medical institution employees to enable forecasting of the institution’s operations in future periods.

## Figures and Tables

**Table 1:** Effective time fractions and time loss by employee category.

Category	Effective Time Fraction (*η*)	Time Loss per Day (%)
Surgeons	0.50	15%
Therapists	0.70	10%
Orthodontists	0.75	10%
Hygienists	0.80	5%
Nurses	0.80	5%
Administrators	0.90	5%
Sanitary Workers	0.90	5%

**Table 2:** Normative and actual workloads of medical institution employees.

ID	Specialty	Workload Share	Normative Workload (Credits)	Real Workload (Credits)
Emp-1	Orthodontist	1.00	1560	2050
Emp-2	Orthodontist	0.75	1170	1160
Emp-3	Surgeon	0.50	420	655
Emp-4	Surgeon	1.00	840	1650
Emp-5	Therapist	0.75	1080	1250
Emp-6	Therapist	1.00	1440	1600
Emp-7	Therapist	0.50	720	600
Emp-8	Therapist	1.00	1440	1450
Emp-9	Hygienist	0.75	1350	1050
Emp-10	Hygienist	1.00	1800	1475
Emp-11	Nurse	0.50	900	532
Emp-12	Nurse	0.25	450	376
Emp-13	Nurse	0.75	1350	928
Emp-14	Nurse	0.50	900	884
Emp-15	Nurse	0.75	1350	884
Emp-16	Nurse	0.50	900	532
Emp-17	Nurse	0.50	900	532
Emp-18	Nurse	0.75	1350	928
Emp-19	Nurse	0.25	450	408
Emp-20	Nurse	0.50	900	908

**Table 3:** Entropy of free time credit distribution.

Category	Number Employees	Total Credits	Entropy	Relative Entropy (%)
All Employees	13	3301	3.299140	89.155354
Doctors	2	130	0.391244	39.124356
Orthodontist	1	10	0.000000	0.000000
Therapist	1	120	0.000000	0.000000
Hygienist	2	625	0.998846	99.884554
Nurse	9	2546	2.809968	88.644624

**Table 4:** Entropy of excessively worked time credit distribution.

Category	Number Employees	Total Credits	Entropy	Relative Entropy (%)
All Employees	7	1883	2.092679	74.542727
Doctors	6	1875	2.061849	79.763207
Orthodontist	1	490	0.000000	0.000000
Surgeon	2	1045	0.768979	76.897934
Therapist	3	340	1.161378	73.274824
Nurse	1	8	0.000000	0.000000

## Data Availability

The data are hosted in an open repository and can be downloaded from there. Link: https://www.researchgate.net/publication/388791228_DC_statistic (accessed on 7 February 2025).

## Appendix A

**Table A1:** Time allocation for medical services by staff category (minutes).

Service	Doctor’s Time (Minutes)	Nurse’s Time (Minutes)	Administrative Time (Minutes)	Sanitary Time (Minutes)
Caries Treatment	25	20	0	5
Pulpitis/Periodontitis Treatment	50	40	0	5
Standard Filling	15	12	0	5
Photopolymer Filling	20	16	0	5
Endodontic Treatment	70	56	0	10
Tooth Extraction (Simple)	15	12	0	5
Wisdom Tooth Extraction (Complex)	50	40	0	5
Dental Implant Placement	110	88	0	10
Root Apex Resection	80	64	0	10
Gum Surgery (Gingivectomy, Recession Repair)	50	40	0	10
Orthodontic Consultation	20	0	0	5
Bracket System Installation	80	64	0	5
Bracket System Adjustment	25	20	0	5
Aligner Fabrication and Installation	50	40	0	5
Orthodontic Retainer Placement	20	16	0	5
Professional Teeth Cleaning (Ultrasound)	35	28	0	5
Fluoride Application	15	12	0	5
Tartar Removal	25	20	0	5
Teeth Whitening (Office-Based)	50	40	0	5
Enamel Polishing	25	20	0	5
Initial Dentist Consultation	15	0	10	0
Panoramic X-ray or CT Scan	0	0	5	0
Dental Treatment Under General Anesthesia	110	88	0	10
